# Effect of Age on the Association between Body Mass Index and All-Cause Mortality: The Ohsaki Cohort Study

**DOI:** 10.2188/jea.JE20090204

**Published:** 2010-09-05

**Authors:** Masato Nagai, Shinichi Kuriyama, Masako Kakizaki, Kaori Ohmori-Matsuda, Yumi Sugawara, Toshimasa Sone, Atsushi Hozawa, Ichiro Tsuji

**Affiliations:** Division of Epidemiology, Department of Public Health and Forensic Medicine, Tohoku University Graduate School of Medicine, Sendai, Japan

**Keywords:** body mass index, mortality, age effect, underweight, obesity

## Abstract

**Background:**

To clarify the effect of age on the association between body mass index (BMI) and all-cause mortality.

**Methods:**

We followed 43 972 Japanese participants aged 40 to 79 years for 12 years. Cox proportional hazards regression analysis was used to estimate hazard ratios (HRs), using the following BMI categories: <18.5 (underweight), 18.5–20.9, 21.0–22.9, 23.0–24.9 (reference), 25.0–27.4, 27.5–29.9, and ≥30.0 kg/m^2^ (obese). Analyses were stratified by age group: middle-aged (40–64 years) vs elderly (65–79 years).

**Results:**

We observed a significantly increased risk of mortality in underweight elderly men: the multivariate HR was 1.26 (0.92–1.73) in middle-aged men and 1.49 (1.26–1.76) in elderly men. In addition, we observed a significantly increased risk of mortality in obese middle-aged men: the multivariate HR was 1.71 (1.17–2.50) in middle-aged men and 1.25 (0.87–1.80) in elderly men. In women, there was an increased risk of mortality irrespective of age group in the underweight: the multivariate HR was 1.46 (0.96–2.22) in middle-aged women and 1.47 (1.19–1.82) in elderly women. There was no excess risk of mortality with age in obese women: the multivariate HR was 1.47 (0.94–2.27) in middle-aged women and 1.26 (0.95–1.68) in elderly women.

**Conclusions:**

As compared with the reference category, obesity was associated with a high mortality risk in middle-aged men, whereas underweight, rather than obesity, was associated with a high mortality risk in elderly men. In women, obesity was associated with a high mortality risk during middle age; underweight was associated with a high mortality risk irrespective of age. The mortality risk due to underweight and obesity may be related to sex and age.

## INTRODUCTION

Epidemiological studies have indicated that the association between body mass index (BMI) and all-cause mortality is dependent upon age.^1^^–^^18^ While almost all studies have agreed that the excess risk of mortality due to obesity attenuates with age,^1^^–^^14^^,^^17^^,^^18^ there is long-standing disagreement regarding the effect of age on the association between underweight and all-cause mortality.^1^^–^^15^ Some studies have shown that the excess risk of mortality due to underweight attenuates with age.^2^^,^^3^^,^^6^^–^^12^^,^^15^ Other studies have indicated that the excess risk of mortality due to underweight increases with age^5^^,^^13^ or remains high irrespective of age.^3^^,^^4^^,^^10^^,^^14^ This inconsistency may be partly due to the inability to control for history of cancer and cardiovascular disease,^4^^,^^6^^,^^7^^,^^10^^,^^12^ and to inadequate adjustment for several other confounders such as cigarette smoking,^14^ alcohol consumption,^7^^–^^9^^,^^12^^,^^14^ physical activity,^7^^–^^10^^,^^12^^,^^14^ and socioeconomic status.^2^^,^^8^^,^^10^^,^^12^^,^^14^^,^^15^ Additionally, several studies failed to include a category for the lowest BMI (<18.5) because of the small proportion of such underweight participants,^1^^,^^3^^,^^8^^,^^9^^,^^13^^–^^15^ or neglected to recruit a study population from the general population.^2^^,^^10^^,^^12^^,^^15^

Serena et al concluded that it is necessary to develop appropriate BMI cut-off points that are country- and ethnic-specific for Asians.^19^ Among 4 Asian studies of the effect of age on the association between BMI and all-cause mortality,^2^^,^^6^^,^^7^^,^^10^ one was conducted in Japan.^10^ In that study, however, multivariate analysis failed to adjust adequately for several confounders. Therefore, the effect of age on the association between underweight and all-cause mortality remains to be clarified.

To further examine the effect of age on the association between BMI and all-cause mortality, we conducted a cohort study among middle-aged and elderly Japanese who were recruited from the general population. We obtained information about their medical history, smoking status, and other possible confounders. In addition, our study overcomes problems in previous studies because we adjusted for several confounders after excluding participants with subclinical disease. We believe that by clarifying the effect of age on the association between BMI and all-cause mortality, it might be possible to improve public health measures by targeting body weight control according to life stage.

## METHODS

### Study cohort

The details of the Ohsaki National Health Insurance (NHI) Cohort Study have been described previously.^20^^–^^22^ Briefly, we delivered a self-administered questionnaire requesting information on various lifestyle habits during the period from October through December 1994 to all NHI beneficiaries aged 40 to 79 years living in the catchment area of the Ohsaki Public Health Center, Miyagi Prefecture, in northeastern Japan. The Ohsaki Public Health Center is a local government agency that provides preventive health services to the residents of 14 municipalities in Miyagi Prefecture. Of 54 996 eligible individuals, 52 029 (95%) responded.

We excluded 776 participants who withdrew from the NHI before 1 January 1995, when we started prospective collection of data on NHI withdrawals. Thus, the study cohort comprised the remaining 51 253 participants. The study protocol was approved by the Ethics Committee of Tohoku University School of Medicine. We considered the return of the self-administered questionnaires signed by the participants to imply their consent to participate in the study.

For the current analysis, we also excluded 1767 participants with a history of cancer, 1384 participants with a history of myocardial infarction, and 997 participants with a history of stroke, because the presence of these diseases at baseline could have affected their BMI. In addition, we excluded 3133 participants who did not provide information about body weight or height. As a result, a total of 43 972 adults (21 038 men and 22 934 women) participated. After 12 years of follow-up, there were 5707 deaths (3685 men and 2022 women).

### Body mass index

The self-administered questionnaire included questions on weight and height. BMI was calculated as weight in kilograms divided by the square of height in meters (kg/m^2^). We used BMI as a measure of total adiposity and divided the participants into groups according to the following BMI categories: <18.5 (underweight), 18.5–20.9, 21.0–22.9, 23.0–24.9, 25.0–27.4, 27.5–29.9, and ≥30.0 kg/m^2^ (obese). These weight categories correspond to the cut-off points proposed by the World Health Organization (WHO), ie, normal BMI range (18.5–24.9 kg/m^2^), grade 1 overweight (25.0–29.9 kg/m^2^), grade 2 overweight (30.0–39.9 kg/m^2^), and grade 3 overweight (≥40.0 kg/m^2^).^23^

We previously evaluated the validity of self-reported weight and height.^22^ Briefly, the weight and height of 14 883 participants, who were a subsample of the cohort, were measured during health examinations in 1995. The Pearson correlation coefficient (*r*) and weighted kappa (κ) for the self-reported values and measured values were *r* = 0.96 (*P* < 0.01) for weight, *r* = 0.93 (*P* < 0.01) for height, and *r* = 0.88 (*P* < 0.01) and κ = 0.72 for BMI. Thus, the self-reported heights and weights in the baseline questionnaire were considered sufficiently valid.

### Follow-up

We followed the participants from 1 January 1995 through 31 December 2006 and recorded any mortality or migration by reviewing data on NHI withdrawals. When a participant withdrew from the NHI system because of death, emigration, or employment, the date of and reason for withdrawal were coded in the NHI withdrawal history files. Because we were unable to obtain subsequent information on participants who withdrew from the NHI because of emigration or employment, we discontinued follow-up of these participants.

The end point was all-cause mortality. Data on the death of participants were based on the death certificates filed at Ohsaki Public Health Center.

The person-years of follow-up were counted for each participant, until either the date of death, withdrawal from the NHI, or the end of the study period, whichever occurred first. The total number of person-years accrued was 440 175.

### Statistical analysis

We used Cox proportional hazards regression analysis to calculate the hazard ratios (HRs) and 95% confidence intervals (CIs) for all-cause mortality according to BMI category, and to adjust for potential confounding factors, using the SAS version 9.1 statistical software package.^24^ To enable detailed examination of the association of BMI and all-cause mortality by WHO categories, the normal weight and overweight categories were divided into 3 and 2 categories, respectively. The BMI category 23.0–24.9 kg/m^2^ was selected as the reference because it is the median of the 7 categories.

Stratified analyses were conducted using 2 age groups: middle-aged participants (40–64 years) and elderly participants (65–79 years). The classification of elderly participants was based on a report by the WHO.^25^ All *P* values were 2-tailed, and a *P* value of <0.05 was considered statistically significant.

The following variables were selected as potential confounding factors: 5-year age group, weight change since age 20 years (loss of ≥10.0 kg, loss of 5.0–9.9 kg, change of less than 5.0 kg, gain of 5.0–9.9 kg, or gain of ≥10.0 kg), education (junior high school or less, high school, or college/university or higher), marital status (married or unmarried), cigarette smoking (never smoker, past smoker, current smoker consuming 1–19 cigarettes per day, or current smoker consuming at least 20 cigarettes per day), alcohol consumption (never drinker, past drinker, or current drinker), time spent walking per day (less than 1 hour or 1 hour or longer), sports and physical exercise time per week (less than 1 hour, 1–2 hours, 3–4 hours, or 5 hours or longer), history of kidney disease (yes or no), and history of liver disease (yes or no). We further adjusted for hypertension and diabetes mellitus in multivariate model 2. Before including the above potential confounders into the multivariate models, we examined interactions between all-cause mortality and all potential confounders through the addition of cross-product terms to the multivariate model. Based on the results of these analyses (data not shown), we included all the above variables into the multivariate models. In addition, we repeated the analyses after excluding the 739 participants who died within 2 years of baseline.

## RESULTS

### Baseline characteristics by BMI category

The baseline characteristics of the study participants according to the 7 BMI categories are shown for middle-aged men (Table 1), elderly men (Table 2), middle-aged women (Table 3), and elderly women (Table 4). Among middle-aged men and women, 2.3% and 2.9%, respectively, were underweight, about 50% of each had a BMI from 21.0 to 24.9 kg/m^2^; 25.7% and 28.5% had a BMI from 25.0 to 29.9 kg/m^2^, and 2.3% and 3.4% were obese, respectively. Among elderly men and women, 5.8% and 5.9%, respectively, were underweight, about half of each had a BMI from 21.0 to 24.9 kg/m^2^; 19.2% and 27.9% had a BMI from 25.0 to 29.9 kg/m^2^, and 1.4% and 4.0% were obese, respectively.

**Table 1.** Baseline characteristics by BMI^a^ category in 13 764 men aged 40–64 years

	BMI (kg/m^2^)							*P* value^b^
	
	<18.5	18.5–20.9	21.0–22.9	23.0–24.9	25.0–27.4	27.5–29.9	≥30.0	
No. of subjects	310	2159	3591	3852	2637	903	312	
Mean age (years) (SD^a^)	54.2 (7.7)	53.0 (8.0)	53.1 (7.8)	53.2 (7.6)	52.8 (7.6)	52.1 (7.4)	52.0 (7.6)	<0.0001
Mean weight (kg) (SD)	48.9 (4.8)	54.6 (4.6)	59.4 (4.7)	64.6 (5.2)	70.3 (5.8)	77.4 (6.3)	86.9 (14.8)	<0.0001
Mean height (cm) (SD)	166.3 (7.6)	164.9 (6.6)	164.0 (6.3)	164.1 (6.2)	164.1 (6.5)	164.8 (6.5)	163.6 (19.1)	<0.0001
Mean BMI (kg/m^2^) (SD)	17.6 (0.9)	20.1 (0.7)	22.1 (0.5)	24.0 (0.6)	26.1 (0.7)	28.5 (0.7)	32.4 (4.7)	<0.0001
Weight change since age 20 years (%)								
≤−10.0 kg	24.4	8.5	4.0	2.8	1.5	2.5	0.7	<0.0001
−9.9 to −5.0 kg	26.8	21.9	14.2	8.0	4.5	3.3	3.0	
−4.9 to +4.9 kg	43.5	61.2	57.2	36.1	15.5	6.6	4.3	
+5.0 to +9.9 kg	4.4	7.1	19.9	32.9	28.1	12.3	7.2	
≥+10.0 kg	1.0	1.3	4.6	20.2	50.4	75.2	84.9	
Education (%)								
Junior high school or less	57.2	56.6	55.8	55.1	52.3	54.2	55.8	NS^a^
High school	35.7	35.9	36.8	37.2	39.0	37.3	35.6	
College/university or higher	7.1	7.5	7.5	7.7	8.7	8.6	8.6	
Marital status (%)								
Married	84.3	87.0	88.8	89.7	88.6	90.4	82.4	0.0072
Unmarried	15.7	13.0	11.2	10.3	11.4	9.6	17.6	
Smoking status (%)								
Never smoker	13.1	13.1	18.2	21.5	24.9	24.9	23.6	<0.0001
Past smoker	16.8	14.1	17.2	19.4	22.9	21.2	19.5	
Current smoker, 1–19 cigarettes/day	23.4	26.6	20.9	19.0	14.7	13.3	13.8	
Current smoker, ≥20 cigarettes/day	46.7	46.3	43.7	40.2	37.5	40.7	43.1	
Alcohol drinking (%)								
Never drinker	20.7	14.7	14.3	14.5	14.7	15.2	19.7	<0.0001
Past drinker	14.4	7.3	7.3	5.9	7.0	5.8	8.9	
Current drinker	64.9	78.0	78.4	79.7	78.3	79.1	71.5	
Time spent walking (%)								
≥1 hour/day	44.3	55.9	53.8	53.0	49.1	46.1	45.3	<0.0001
<1 hour/day	55.8	44.1	46.2	47.0	50.9	53.9	54.7	
Sports and physical exercise (%)								
≥5 hours/week	2.4	5.8	5.4	5.8	4.5	4.4	5.9	0.0367
3–4 hours/week	3.8	4.1	4.7	5.2	5.0	4.8	3.0	
1–2 hours/week	11.9	12.1	14.2	14.1	14.3	15.8	12.9	
<1 hour/week	81.9	78.1	75.7	75.0	76.2	75.0	78.2	
History of hypertension (%)								
Yes	10.0	12.6	14.6	17.2	21.2	26.8	25.3	<0.0001
No	90.0	87.4	85.4	82.8	78.8	73.2	74.7	
History of diabetes (%)								
Yes	6.1	4.5	5.9	5.9	6.6	7.3	9.6	0.0015
No	93.9	95.6	94.2	94.1	93.4	92.7	90.4	
History of kidney disease (%)								
Yes	4.5	4.3	3.3	3.0	3.5	4.7	2.6	NS
No	95.5	95.7	96.7	97.0	96.6	95.4	97.4	
History of liver disease (%)								
Yes	6.1	6.4	6.2	7.0	7.4	7.6	8.0	NS
No	93.9	93.7	93.8	93.0	92.6	92.4	92.0	

^a^BMI, body mass index; SD, standard deviation; NS, not significant.

^b^*P* values were calculated by using the chi-square test (for categorical variables) or ANOVA (for continuous variables).

**Table 2.** Baseline characteristics by BMI^a^ category in 7274 men aged 65–79 years

	BMI (kg/m^2^)							*P* value^b^
	
	<18.5	18.5–20.9	21.0–22.9	23.0–24.9	25.0–27.4	27.5–29.9	≥30.0	
No. of subjects	422	1518	2026	1805	1089	310	104	
Mean age (years) (SD^a^)	71.5 (4.2)	70.4 (4.2)	70.2 (4.0)	69.9 (4.0)	69.6 (3.9)	69.5 (3.8)	69.9 (4.3)	<0.0001
Mean weight (kg) (SD)	47.1 (4.9)	52.0 (4.4)	56.5 (4.7)	61.6 (5.0)	66.7 (5.4)	72.4 (6.5)	81.0 (21.6)	<0.0001
Mean height (cm) (SD)	164.5 (8.0)	161.1 (6.4)	160.1 (6.5)	160.4 (6.3)	159.9 (6.2)	159.6 (7.2)	153.8 (13.7)	<0.0001
Mean BMI (kg/m^2^) (SD)	17.4 (1.0)	20.0 (0.7)	22.0 (0.6)	23.9 (0.6)	26.0 (0.7)	28.4 (0.6)	34.3 (8.1)	<0.0001
Weight change since age 20 years (%)								
≤−10.0 kg	55.0	29.9	13.5	6.9	3.1	3.1	4.0	<0.0001
−9.9 to −5.0 kg	28.9	37.1	30.5	16.6	8.4	5.9	5.0	
−4.9 to +4.9 kg	15.4	29.9	43.6	43.0	26.1	13.8	12.0	
+5.0 to +9.9 kg	0.8	2.3	9.7	22.1	29.1	19.3	8.0	
≥+10.0 kg	0.0	0.8	2.7	11.4	33.4	57.9	71.0	
Education (%)								
Junior high school or less	69.4	73.9	75.4	72.1	73.2	72.9	69.6	0.0107
High school	20.9	19.4	18.5	19.2	20.9	22.4	24.5	
College/university or higher	9.8	6.7	6.1	8.8	6.0	4.7	5.9	
Marital status (%)								
Married	89.8	90.3	90.0	89.2	91.7	91.4	89.6	NS^a^
Unmarried	10.2	9.7	10.0	10.9	8.3	8.6	10.4	
Smoking status (%)								
Never smoker	12.7	14.0	14.6	18.5	18.6	23.4	29.4	<0.0001
Past smoker	32.7	31.7	34.9	38.9	43.1	42.3	35.9	
Current smoker, 1–19 cigarettes/day	34.8	30.7	29.2	24.5	21.0	15.7	18.5	
Current smoker, ≥20 cigarettes/day	19.8	23.6	21.4	18.1	17.3	18.5	16.3	
Alcohol drinking (%)								
Never drinker	21.3	19.3	19.9	19.2	18.4	20.1	20.8	<0.0001
Past drinker	24.0	18.1	15.0	14.0	14.9	12.3	18.8	
Current drinker	54.8	62.6	65.1	66.7	66.7	67.6	60.4	
Time spent walking (%)								
≥1 hour/day	36.9	47.1	46.0	43.0	37.6	41.7	34.8	<0.0001
<1 hour/day	63.1	52.9	54.0	57.0	62.4	58.3	65.2	
Sports and physical exercise (%)								
≥5 hours/week	16.2	15.4	17.0	17.4	15.3	15.4	10.7	NS
3–4 hours/week	9.2	9.8	10.4	10.4	11.4	8.8	7.1	
1–2 hours/week	14.8	17.0	18.9	17.6	20.4	24.2	26.2	
<1 hour/week	59.9	57.9	53.7	54.5	52.9	51.7	56.0	
History of hypertension (%)								
Yes	21.6	27.8	32.8	38.2	43.3	48.4	52.9	<0.0001
No	78.4	72.2	67.2	61.8	56.7	51.6	47.1	
History of diabetes (%)								
Yes	6.2	6.7	9.2	9.7	9.7	12.9	11.5	0.0010
No	93.8	93.3	90.8	90.3	90.3	87.1	88.5	
History of kidney disease (%)								
Yes	5.0	4.0	3.4	4.5	3.5	2.9	3.9	NS
No	95.0	96.1	96.6	95.5	96.5	97.1	96.2	
History of liver disease (%)								
Yes	8.3	7.7	5.8	5.7	6.5	7.4	11.5	0.0284
No	91.7	92.3	94.2	94.4	93.5	92.6	88.5	

^a^BMI, body mass index; SD, standard deviation; NS, not significant.

^b^*P* values were calculated by using the chi-square test (for categorical variables) or ANOVA (for continuous variables).

**Table 3.** Baseline characteristics by BMI^a^ category in 14 457 women aged 40–64 years

	BMI (kg/m^2^)							*P* value^b^
	
	<18.5	18.5–20.9	21.0–22.9	23.0–24.9	25.0–27.4	27.5–29.9	≥30.0	
No. of subjects	425	2135	3521	3770	2890	1227	489	
Mean age (years) (SD^a^)	54.1 (7.7)	53.5 (7.7)	53.9 (7.5)	54.4 (7.2)	55.6 (6.9)	55.4 (6.8)	54.9 (7.0)	<0.0001
Mean weight (kg) (SD)	42.1 (4.1)	47.3 (3.7)	51.4 (3.7)	55.9 (4.0)	60.4 (4.4)	65.6 (4.9)	73.0 (11.0)	<0.0001
Mean height (cm) (SD)	154.8 (7.7)	153.6 (5.4)	152.7 (5.2)	152.6 (5.2)	152.0 (5.2)	151.5 (5.5)	149.6 (8.6)	<0.0001
Mean BMI (kg/m^2^) (SD)	17.5 (0.9)	20.0 (0.7)	22.0 (0.6)	24.0 (0.6)	26.1 (0.7)	28.5 (0.7)	32.6 (4.6)	<0.0001
Weight change since age 20 years (%)								
≤−10.0 kg	19.1	8.2	3.2	1.2	0.4	0.9	0.9	<0.0001
−9.9 to −5.0 kg	31.4	22.5	14.0	5.6	2.6	1.3	1.5	
−4.9 to +4.9 kg	46.8	58.6	52.7	35.6	15.0	7.1	4.3	
+5.0 to +9.9 kg	2.2	9.5	24.3	36.9	33.7	17.4	6.1	
≥+10.0 kg	0.5	1.3	5.8	20.8	48.3	73.4	87.2	
Education (%)								
Junior high school or less	49.4	42.8	45.7	49.2	55.1	58.8	63.9	<0.0001
High school	40.2	45.0	43.3	41.6	37.8	34.1	31.1	
College/university or higher	10.4	12.2	11.0	9.3	7.0	7.1	5.0	
Marital status (%)								
Married	74.4	81.7	83.6	84.8	84.1	84.1	81.1	<0.0001
Unmarried	25.6	18.3	16.4	15.2	15.9	15.9	18.9	
Smoking status (%)								
Never smoker	79.4	82.8	88.8	90.1	89.1	88.0	87.9	<0.0001
Past smoker	2.3	2.5	1.8	2.0	2.3	2.4	2.1	
Current smoker, 1–19 cigarettes/day	12.4	9.6	6.4	5.3	6.2	5.7	5.1	
Current smoker, ≥20 cigarettes/day	5.9	5.1	3.0	2.6	2.5	3.8	4.9	
Alcohol drinking (%)								
Never drinker	66.9	64.7	68.4	68.0	68.2	69.2	64.8	0.0002
Past drinker	6.4	5.1	3.4	3.6	4.4	5.6	8.5	
Current drinker	26.7	30.2	28.3	28.3	27.4	25.2	26.7	
Time spent walking (%)								
≥1 hour/day	41.0	47.5	46.8	47.9	45.2	39.9	39.4	<0.0001
<1 hour/day	59.0	52.5	53.2	52.1	54.8	60.1	60.6	
Sports and physical exercise (%)								
≥5 hours/week	3.6	3.5	4.4	3.7	3.9	3.0	3.6	NS^a^
3–4 hours/week	4.1	4.3	4.9	5.1	5.1	4.5	3.4	
1–2 hours/week	14.0	14.6	14.1	14.7	16.4	14.7	11.7	
<1 hour/week	78.4	77.6	76.6	76.5	74.6	77.8	81.4	
History of hypertension (%)								
Yes	10.6	11.0	15.2	20.7	28.8	35.6	41.3	<0.0001
No	89.4	89.0	84.8	79.3	71.2	64.4	58.7	
History of diabetes (%)								
Yes	3.3	3.4	3.2	3.5	4.5	4.5	6.1	0.0043
No	96.7	96.6	96.9	96.6	95.5	95.5	93.9	
History of kidney disease (%)								
Yes	6.4	5.3	3.9	3.2	2.7	3.9	4.9	<0.0001
No	93.7	94.7	96.1	96.8	97.3	96.1	95.1	
History of liver disease (%)								
Yes	5.2	3.2	3.6	3.9	3.7	4.5	5.7	NS
No	94.8	96.8	96.4	96.1	96.3	95.5	94.3	

^a^BMI, body mass index; SD, standard deviation; NS, not significant.

^b^*P* values were calculated by using the chi-square test (for categorical variables) or ANOVA (for continuous variables).

**Table 4.** Baseline characteristics by BMI^a^ category in 8477 women aged 65–79 years

	BMI (kg/m^2^)							*P* value^b^
	
	<18.5	18.5–20.9	21.0–22.9	23.0–24.9	25.0–27.4	27.5–29.9	≥30.0	
No. of subjects	503	1383	1977	1906	1666	702	340	
Mean age (years) (SD^a^)	72.0 (4.3)	70.9 (4.3)	70.4 (4.2)	70.0 (4.0)	70.0 (4.0)	70.0 (4.1)	70.0 (4.0)	<0.0001
Mean weight (kg) (SD)	39.6 (4.5)	44.6 (3.7)	48.8 (3.7)	53.6 (4.0)	58.1 (4.7)	62.5 (5.2)	68.7 (12.0)	<0.0001
Mean height (cm) (SD)	151.9 (8.8)	149.5 (5.8)	148.8 (5.3)	149.3 (5.3)	149.0 (5.6)	147.8 (5.9)	144.6 (10.3)	<0.0001
Mean BMI (kg/m^2^) (SD)	17.2 (1.2)	19.9 (0.7)	22.0 (0.6)	24.0 (0.6)	26.1 (0.7)	28.6 (0.7)	33.0 (5.6)	<0.0001
Weight change since age 20 years (%)								
≤−10.0 kg	41.9	22.7	12.0	4.5	3.3	1.5	1.3	<0.0001
−9.9 to −5.0 kg	33.3	35.1	25.4	16.1	8.7	4.5	2.6	
−4.9 to +4.9 kg	22.7	36.9	46.3	39.0	21.6	15.3	6.2	
+5.0 to +9.9 kg	1.8	4.9	12.7	26.6	29.9	22.7	13.1	
≥+10.0 kg	0.2	0.3	3.6	13.8	36.5	56.0	76.8	
Education (%)								
Junior high school or less	65.7	68.8	68.4	67.1	72.6	75.2	82.1	<0.0001
High school	28.6	26.4	25.0	26.2	22.1	19.2	14.7	
College/university or higher	5.7	4.8	6.6	6.7	5.2	5.6	3.3	
Marital status (%)								
Married	59.8	61.7	62.9	62.8	63.9	65.5	62.0	NS^a^
Unmarried	40.2	38.4	37.1	37.2	36.1	34.5	38.0	
Smoking status (%)								
Never smoker	84.1	90.0	90.0	91.1	92.3	91.4	89.8	0.0016
Past smoker	4.1	2.6	3.0	3.6	2.9	3.0	5.1	
Current smoker, 1–19 cigarettes/day	10.5	6.3	6.1	4.8	3.9	4.7	3.9	
Current smoker, ≥20 cigarettes/day	1.3	1.1	1.0	0.5	0.8	1.0	1.2	
Alcohol drinking (%)								
Never drinker	82.0	81.1	81.8	81.4	82.0	78.3	80.4	NS
Past drinker	4.6	4.5	4.9	4.4	3.4	4.8	5.8	
Current drinker	13.5	14.4	13.3	14.2	14.6	16.9	13.8	
Time spent walking (%)								
≥1 hour/day	34.3	40.4	39.8	38.7	35.5	34.7	28.5	0.0002
<1 hour/day	65.7	59.6	60.2	61.3	64.5	65.3	71.5	
Sports and physical exercise (%)								
≥5 hours/week	4.6	8.4	6.9	9.2	8.5	7.2	9.7	0.0003
3–4 hours/week	6.7	7.6	8.5	8.1	8.1	7.8	6.6	
1–2 hours/week	12.7	14.9	19.3	19.4	18.2	18.1	13.1	
<1 hour/week	76.0	69.0	65.3	63.3	65.2	66.9	70.7	
History of hypertension (%)								
Yes	24.7	29.9	35.0	39.8	45.8	50.7	54.7	<0.0001
No	75.4	70.1	65.0	60.2	54.2	49.3	45.3	
History of diabetes (%)								
Yes	5.8	6.1	8.7	7.5	8.2	9.0	12.9	0.0004
No	94.2	93.9	91.4	92.6	91.8	91.0	87.1	
History of kidney disease (%)								
Yes	4.4	4.3	4.5	4.0	5.0	4.4	2.1	NS
No	95.6	95.7	95.5	96.0	95.0	95.6	97.9	
History of liver disease (%)								
Yes	4.0	4.8	5.5	5.0	3.8	4.6	6.2	NS
No	96.0	95.2	94.5	95.0	96.2	95.4	93.8	

^a^BMI, body mass index; SD, standard deviation; NS, not significant.

^b^*P* values were calculated by using the chi-square test (for categorical variables) or ANOVA (for continuous variables).

In men, mean age decreased linearly with an increase in BMI category. In women, middle-aged women with a BMI from 25.0 to 27.4 kg/m^2^ and elderly women who were underweight were oldest. The proportions of men and women who had lost ≥5 kg of body weight since age 20 years decreased with increasing BMI category. Participants with the highest level of education were middle-aged men with a BMI from 25.0 to 27.4 kg/m^2^, middle-aged women with a BMI from 18.5 to 20.9 kg/m^2^, and underweight elderly men and women. The proportions of unmarried men and women were higher among those who were underweight and obese. The proportions of men and women who were current smokers decreased with increasing BMI. The proportions of men and women who had never drunk alcohol were highest in the underweight, with the exception of middle-aged women. Underweight and obese men and women were less likely to walk 1 hour or longer per day and to participate in <1 hour of sports or physical exercise per week. The proportions of men and women who had histories of hypertension and diabetes increased with an increase in BMI category. The proportions of middle-aged men and elderly women who had histories of kidney disease and liver disease did not significantly differ across BMI categories. The proportions of participants with histories of liver disease and kidney disease were highest among elderly obese men and underweight middle-aged women, respectively.

### All-cause mortality by BMI category

Table 5 (for men) and Table 6 (for women) show person-year totals, numbers of all-cause deaths, and HRs of all-cause mortality with 95% CIs according to BMI category and age group.

**Table 5.** HRs^a^ and 95% CIs^a^ of all-cause mortality in 21 038 men by BMI^a^ category, stratified by age group

	BMI						
	
	<18.5	18.5–20.9	21.0–22.9	23.0–24.9	25.0–27.4	27.5–29.9	≥30.0
Total							
No.	732	3677	5617	5657	3726	1213	416
Person-years	6282	35 339	55 681	57 157	37 954	12 484	4162
No. of deaths	270	805	1004	861	513	165	67
Mortality rate^b^	43.0	22.8	18.0	15.1	13.5	13.2	16.1
Age-smoking-adjusted HRs	2.78 (2.42–3.18)	1.49 (1.35–1.64)	1.19 (1.09–1.22)	1.00 (reference)	0.90 (0.81–1.00)	0.90 (0.76–1.06)	1.11 (0.87–1.43)
Multivariate HRs1^c^	1.42 (1.23–1.65)	1.10 (0.99–1.22)	1.04 (0.95–1.14)	1.00 (reference)	1.01 (0.90–1.13)	1.10 (0.92–1.31)	1.44 (1.11–1.87)
Multivariate HRs2^d^	1.52 (1.31–1.76)	1.14 (1.03–1.26)	1.06 (0.96–1.16)	1.00 (reference)	1.00 (0.89–1.12)	1.06 (0.89–1.26)	1.39 (1.07–1.81)
Multivariate HRs3^e^	1.35 (1.15–1.59)	1.06 (0.95–1.19)	1.01 (0.92–1.12)	1.00 (reference)	0.99 (0.88–1.12)	1.05 (0.87–1.26)	1.42 (1.07–1.88)

40–64 y							
No.	310	2159	3591	3852	2637	903	312
Person-years	3053	21 992	36 885	40 026	27 421	9425	3221
No. of deaths	47	224	340	305	211	75	33
Mortality rate^b^	15.4	10.2	9.2	7.6	7.7	8.0	10.2
Age-smoking-adjusted HRs	1.76 (1.29–2.39)	1.25 (1.05–1.49)	1.17 (1.01–1.37)	1.00 (reference)	1.07 (0.90–1.28)	1.17 (0.91–1.51)	1.54 (1.08–2.21)
Multivariate HRs1^c^	1.26 (0.92–1.73)	1.07 (0.89–1.28)	1.11 (0.95–1.30)	1.00 (reference)	1.14 (0.95–1.37)	1.27 (0.97–1.66)	1.71 (1.17–2.50)
Multivariate HRs2^d^	1.32 (0.96–1.82)	1.09 (0.91–1.31)	1.12 (0.96–1.32)	1.00 (reference)	1.12 (0.93–1.35)	1.22 (0.93–1.60)	1.64 (1.12–2.40)
Multivariate HRs3^e^	1.24 (0.87–1.78)	1.11 (0.92–1.35)	1.13 (0.95–1.34)	1.00 (reference)	1.16 (0.96–1.41)	1.20 (0.90–1.61)	1.62 (1.07–2.45)
65–79 y							
No.	422	1518	2026	1805	1089	310	104
Person-years	3229	13 347	18 796	17 131	10 533	3059	941
No. of deaths	223	581	664	556	302	90	34
Mortality rate^b^	69.1	43.5	35.3	32.5	28.7	29.4	36.1
Age-smoking-adjusted HRs	1.88 (1.61–2.20)	1.26 (1.12–1.41)	1.06 (0.94–1.18)	1.00 (reference)	0.91 (0.79–1.05)	0.96 (0.77–1.19)	1.21 (0.86–1.71)
Multivariate HRs1^c^	1.49 (1.26–1.76)	1.11 (0.98–1.26)	1.01 (0.90–1.14)	1.00 (reference)	0.94 (0.81–1.09)	1.01 (0.80–1.27)	1.25 (0.87–1.80)
Multivariate HRs2^d^	1.59 (1.35–1.89)	1.16 (1.03–1.32)	1.03 (0.92–1.16)	1.00 (reference)	0.93 (0.80–1.07)	0.97 (0.77–1.23)	1.23 (0.86–1.76)
Multivariate HRs3^e^	1.48 (1.23–1.78)	1.09 (0.96–1.25)	0.98 (0.87–1.11)	1.00 (reference)	0.88 (0.76–1.03)	0.91 (0.71–1.17)	1.22 (0.83–1.79)

^a^HR, hazard ratio; CI, confidence interval; BMI, body mass index.

^b^Mortality rate was defined as number of deaths per 1000 person-years.

^c^Multivariate HRs1 were adjusted for age in 5-year categories; weight change since age 20 years (loss of 10.0 kg or more, loss of 5.0–9.9 kg, change of less than ±5.0 kg, gain of 5.0–9.9 kg, or gain of 10.0 kg or more); education (junior high school or less, high school, or college/university or higher); marital status (married or unmarried); cigarette smoking (never smoker, past smoker, current smoker consuming 1–19 cigarettes per day, or current smoker consuming at least 20 cigarettes per day); alcohol drinking (never drinker, past drinker, or current drinker); time spent walking per day (less than 1 hour, or 1 hour or longer); sports and physical exercise time per week (less than 1 hour, 1–2 hours, 3–4 hours, or 5 hours or longer); history of kidney disease (yes or no); history of liver disease (yes or no).

^d^Multivariate HRs2 were further adjusted for history of hypertension (yes or no) and history of diabetes (yes or no).

^e^Multivariate HRs3 excluded from multivariate HRs2 the 473 men who died within the 2 years after baseline.

**Table 6.** HRs^a^ and 95% CIs^a^ of all-cause mortality in 22 934 women by BMI^a^ category, stratified by age group

	BMI						
	
	<18.5	18.5–20.9	21.0–22.9	23.0–24.9	25.0–27.4	27.5–29.9	≥30.0
Total							
No.	928	3518	5498	5676	4556	1929	829
Person-years	9011	34 782	55 716	57 537	46 281	19 477	8313
No. of deaths	174	371	451	415	357	159	95
Mortality rate^b^	19.3	10.7	8.1	7.2	7.7	8.2	11.4
Age-smoking-adjusted HRs	2.66 (2.23–3.18)	1.48 (1.28–1.70)	1.12 (0.98–1.28)	1.00 (reference)	1.07 (0.93–1.23)	1.13 (0.94–1.36)	1.59 (1.27–1.99)
Multivariate HRs1^c^	1.49 (1.24–1.80)	1.15 (0.99–1.33)	0.99 (0.87–1.14)	1.00 (reference)	1.03 (0.89–1.19)	1.07 (0.89–1.30)	1.33 (1.05–1.69)
Multivariate HRs2^d^	1.58 (1.31–1.91)	1.19 (1.03–1.38)	1.01 (0.88–1.15)	1.00 (reference)	1.00 (0.87–1.16)	1.04 (0.85–1.26)	1.24 (0.97–1.57)
Multivariate HRs3^e^	1.44 (1.17–1.78)	1.18 (1.01–1.38)	1.02 (0.88–1.18)	1.00 (reference)	1.06 (0.91–1.24)	1.09 (0.88–1.34)	1.37 (1.07–1.77)

40–64 y							
No.	425	2135	3521	3770	2890	1227	489
Person-years	4416	21 274	35 734	38 262	29 435	12 484	4999
No. of deaths	32	92	137	128	104	49	28
Mortality rate^b^	7.2	4.3	3.8	3.3	3.5	3.9	5.6
Age-smoking-adjusted HRs	2.10 (1.43–3.10)	1.30 (0.99–1.70)	1.16 (0.91–1.48)	1.00 (reference)	0.99 (0.76–1.28)	1.11 (0.80–1.54)	1.59 (1.06–2.39)
Multivariate HRs1^c^	1.46 (0.96–2.22)	1.10 (0.82–1.47)	1.09 (0.85–1.40)	1.00 (reference)	1.00 (0.77–1.31)	1.06 (0.75–1.51)	1.47 (0.94–2.27)
Multivariate HRs2^d^	1.55 (1.02–2.36)	1.14 (0.85–1.52)	1.10 (0.86–1.41)	1.00 (reference)	0.98 (0.75–1.28)	1.01 (0.71–1.43)	1.38 (0.89–2.14)
Multivariate HRs3^e^	1.78 (1.13–2.81)	1.36 (1.00–1.86)	1.21 (0.92–1.59)	1.00 (reference)	1.02 (0.76–1.36)	0.99 (0.68–1.45)	1.32 (0.82–2.15)
65–79 y							
No.	503	1383	1977	1906	1666	702	340
Person-years	4595	13 508	19 982	19 275	16 845	6994	3314
No. of deaths	142	279	314	287	253	110	67
Mortality rate^b^	30.9	20.7	15.7	14.9	15.0	15.7	20.2
Age-smoking-adjusted HRs	1.67 (1.37–2.05)	1.26 (1.07–1.48)	1.00 (0.85–1.17)	1.00 (reference)	1.02 (0.86–1.21)	1.07 (0.86–1.34)	1.33 (1.02–1.73)
Multivariate HRs1^c^	1.47 (1.19–1.82)	1.14 (0.96–1.36)	0.95 (0.81–1.12)	1.00 (reference)	1.04 (0.87–1.24)	1.07 (0.85–1.35)	1.26 (0.95–1.68)
Multivariate HRs2^d^	1.56 (1.26–1.93)	1.19 (1.00–1.41)	0.96 (0.82–1.13)	1.00 (reference)	1.01 (0.85–1.21)	1.04 (0.83–1.31)	1.17 (0.88–1.55)
Multivariate HRs3^e^	1.45 (1.15–1.83)	1.17 (0.97–1.40)	0.96 (0.81–1.15)	1.00 (reference)	1.04 (0.86–1.25)	1.07 (0.83–1.37)	1.24 (0.92–1.68)

^a^HR, hazard ratio; CI, confidence interval; BMI, body mass index.

^b^Mortality rate was defined as number of deaths per 1000 person-years.

^c^Multivariate HRs1 were adjusted for age in 5-year categories; weight change since age 20 years (loss of 10.0 kg or more, loss of 5.0–9.9 kg, change of less than ±5.0 kg, gain of 5.0–9.9 kg, or gain of 10.0 kg or more); education (junior high school or less, high school, or college/university or higher); marital status (married or unmarried); cigarette smoking (never smoker, past smoker, current smoker consuming 1–19 cigarettes per day, or current smoker consuming at least 20 cigarettes per day); alcohol drinking (never drinker, past drinker, or current drinker); time spent walking per day (less than 1 hour, or 1 hour or longer); sports and physical exercise time per week (less than 1 hour, 1–2 hours, 3–4 hours, or 5 hours or longer); history of kidney disease (yes or no); history of liver disease (yes or no).

^d^Multivariate HRs2 were further adjusted for history of hypertension (yes or no) and history of diabetes (yes or no).

^e^Multivariate HRs3 excluded from multivariate HRs2 the 266 women who died within the 2 years after baseline.

In men, we observed significantly increased risks of mortality in the underweight and obese: the model 1 multivariate HRs (95% CI) were 1.42 (1.23–1.65) and 1.44 (1.11–1.87), respectively. After stratification by age group, we observed a significantly increased risk of mortality in elderly underweight men: the model 1 multivariate HRs were 1.26 (0.92–1.73) in middle-aged men and 1.49 (1.26–1.76) in elderly men. There was also a significantly increased risk of mortality in middle-aged obese men: the model 1 multivariate HRs were 1.71 (1.17–2.50) in middle-aged men and 1.25 (0.87–1.80) in elderly men.

In women, we observed significantly increased risks of mortality in the underweight and obese: the model 1 multivariate HRs were 1.49 (1.24–1.80) and 1.33 (1.05–1.69), respectively. After stratification by age group, we observed an increased risk of mortality irrespective of age group in the underweight category: the model 1 multivariate HRs were 1.46 (0.96–2.22) in middle-aged women and 1.47 (1.19–1.82) in elderly women. However, we did not observe an excess risk of mortality with age in the obese: the model 1 multivariate HRs were 1.47 (0.94–2.27) in middle-aged women and 1.26 (0.95–1.68) in elderly women.

The inclusion of covariates for histories of hypertension and diabetes (model 2) attenuated the HR in adults with a BMI ≥25.0 kg/m^2^ and increased the HR in those with a BMI <23.0 kg/m^2^. However, model 2 multivariate HRs were similar to model 1 HRs. After the exclusion of participants who died during the first 2 years of follow-up (model 3), multivariate HRs were similar to model 2 HRs in men and obese women. In underweight women, however, there was no excess risk of mortality with age: the model 3 multivariate HRs were 1.78 (1.13–2.81) in middle-aged adults and 1.45 (1.15–1.83) in elderly adults.

We also calculated model 1 multivariate HRs after changing the reference category to 18.5 ≤ BMI ≤ 24.9 kg/m^2^ from 23.0 ≤ BMI ≤ 24.9 kg/m^2^ (model 4). The HRs were similar to model 1 HRs: the model 4 multivariate HRs in underweight men were 1.18 (0.88–1.60) in middle-aged men and 1.42 (1.26–1.76) in elderly men, in obese men they were 1.64 (1.13–2.38) in middle-aged men and 1.25 (0.87–1.80) in elderly men, in underweight women they were 1.38 (0.94–1.99) in middle-aged women and 1.43 (1.19–1.71) in elderly women, and in obese women they were 1.41 (0.92–2.16) in middle-aged women and 1.25 (0.95–1.64) in elderly women.

## DISCUSSION

The present results indicate that the mortality risk associated with underweight and obesity might be dependent upon sex and age group. We noted significant increased risks of mortality only in middle-aged obese men and elderly underweight men. In women, there was no significant excess risk of mortality with age in the obese, and no significant increased risk of mortality, irrespective of age group, in the underweight.

We considered several important confounding factors: cigarette smoking, alcohol consumption, and physical activity are major confounding factors associated with both BMI and mortality.^1^^–^^15^^,^^17^^,^^18^ We also considered education level and marital status as potential confounding factors, as in past studies.^1^^,^^3^^,^^4^^,^^6^^,^^7^^,^^9^^,^^11^^,^^17^ Furthermore, the presence of subclinical disease or a history of illness could induce weight loss and increase the risk of death.^1^^–^^4^^,^^8^^,^^9^^,^^11^^,^^14^^,^^15^^,^^17^^,^^18^ To eliminate any effect of medical history, we excluded participants with a history of cancer, myocardial infarction, or stroke, and adjusted for weight change since age 20 years, history of kidney disease, and history of liver disease, in multivariate analysis.

Multivariate adjustment attenuated the HR estimates associated with a BMI of 27.5–29.9 or ≥30.0 kg/m^2^ in women, but not in men. No single covariate resulted in significant attenuation, although an increase in body weight of 5 kg or more since age 20 years, current drinking, and ≥1 hour physical activity per week attenuated hazard ratios. In contrast, a decrease in body weight of 5 kg or less since age 20 years, past drinking, being unmarried, <1 hour spent walking per day, and histories of kidney disease and liver disease significantly increased HRs in men.

Almost all previous studies agree that the excess risk of mortality due to obesity decreases with age,^1^^–^^14^^,^^17^^,^^18^ and our results accord with this. In underweight adults, the results of past studies have been inconsistent.^1^^–^^15^ Our results are in agreement with 2 of 14 studies of men,^5^^,^^13^ and 4 of 13 studies of women.^3^^,^^4^^,^^10^^,^^14^

In Japan, Matsuo et al reported the effect of age on the association between BMI and all-cause mortality.^10^ Their findings agree with ours, except for underweight men. They adjusted only for age, alcohol intake, and smoking status in multivariate analysis; however, physical activity and socioeconomic status have also been identified as confounding factors for the risk of all-cause mortality.^1^^–^^15^^,^^17^^,^^18^ Although their result differ from ours for underweight men, our study was more careful in adjusting for physical activity, socioeconomic status, weight change since age 20 years, marital status, and histories of kidney disease and liver disease.

Development of measures to address underweight has been slower than for obesity. However, Grabowski et al and Sergi et al showed that a low BMI in elderly adults was a predictor of mortality.^26^^,^^27^ Okoro et al found that underweight was associated with subsequent disability in elderly adults.^28^ Our study also found that underweight is associated with a high mortality risk in elderly men and women, irrespective of age group.

A major strength of the present study was that the participants were recruited from the general Japanese population. According to the Global Database on Body Mass Index of the WHO, the prevalence of underweight participants is higher in Japan (10%–20%) than in Western populations (0%–5%). Therefore, the Japanese population is one of the best in which to examine the excess risk of mortality due to underweight.

Several limitations of our study should be considered. First, although BMI has been accepted as satisfactory index of underweight and obesity, it cannot be used to identify distributions of fat and muscle tissue. Second, we used self-reported BMI at baseline. Niedhammer et al showed that there is a systematic bias in self-reported weight and height.^29^ However, we previously evaluated the validity of self-reported BMI, and demonstrated a high correlation and appropriate agreement between self-reported BMI and measured BMI in a subsample of 14 883 participants (*r* = 0.88, κ = 0.72). We consider this bias to be a nondifferential misclassification that is not dependent upon all-cause death. This misclassification weakens the true association toward the null. Third, as a result of stratification by age group, there was a possibility of beta error because of inadequate numbers of participants and events. Finally, there is a possibility of residual confounding by physical activity.

In summary, obesity increases mortality risk in middle-aged men, whereas underweight, rather than obesity, is associated with high mortality risk in elderly men. In women, obesity increases mortality risk in middle age, and underweight increases mortality risk irrespective of age. Although there was no significant interaction by age group or sex, the mortality risks associated with underweight and obesity may nevertheless be dependent on sex and age group.
